# Development of a Versatile Half-Strip Lateral Flow Assay toward the Detection of Rift Valley Fever Virus Antibodies

**DOI:** 10.3390/diagnostics12112664

**Published:** 2022-11-02

**Authors:** Thulaganyo Domfe, Zikhona Njengele-Tetyana, Nikiwe Mhlanga, Phumlani Tetyana, Amanda Skepu, Jane Catherine Ngila, Lucky Mashudu Sikhwivhilu

**Affiliations:** 1 Department of Chemical Sciences, University of Johannesburg, Doornfontein, Johannesburg 2050, South Africa; 2 Advanced Materials Division, DSI/Mintek NIC, Mintek, 200 Malibongwe Drive, Randburg 2194, South Africa; 3 Next Generation Health, Council for Scientific and Industrial Research, Meiring Naude Road, Brummeria, Pretoria 0001, South Africa; 4 The African Academy of Science, 8 Miotoni Lane, Karen, Nairobi P.O. Box 24916-00502, Kenya; 5 Department of Chemistry, Faculty of Science, Engineering and Agriculture, Thohoyandou 0950, South Africa

**Keywords:** Rift Valley fever virus, protein expression, diagnostic assay, enzyme-linked immunosorbent assay

## Abstract

Rift Valley fever (RVF) is a mosquito-borne zoonotic disease that is caused by the Rift Valley fever virus (RVFV); *Bunyaviridae*: Phlebovirus. RVF disease can affect several different species, including ruminants, camels and humans and thus present a dual threat to public health and livestock food production in endemic regions. In livestock, the RVFV infection is characterised by an acute hepatitis, abortion and high mortality rates in new-born animals. The current RVF diagnostic techniques have shown good sensitivity. However, they require extensive sample processing and complex instrumentation. Owing to speed, low cost, ease of use, and most importantly, the ability to diagnose diseases at sites where they are managed, lateral flow immunoassays (LFIA) are the most widely used point-of-care (POC) tools for disease diagnosis. In this study, a lateral flow assay (LFA) device that is able to detect antibodies against RVFV, with a minimum detectable concentration of 0.125 mg/mL, was successfully developed. The LFA also successfully detected RVFV antibodies in reference RVFV sera. Protein A (ProA), which has the ability to bind immunoglobulins from different species, was used in the detection probe, giving the developed RVFV LFA potential for multi-species diagnosis.

## 1. Introduction

The Rift Valley fever virus (RVFV) is a spherical-shaped, enveloped virus with a negative-sense single-stranded RNA genome made up of three segments designated L, M and S of negative or ambisense polarity [1]. These segments encode the RNA-dependent RNA polymerase L, N protein and the glycoproteins, respectively. RVFV has been a long-recognised veterinary disease of livestock in endemic regions. It can result in dire economic impact for the affected regions causing high morbidity and mortality in domesticated livestock (i.e., goats, cattle and sheep) [2]. RVFV mortality rate in humans is estimated at 0.5–2.0% overall [3].

Since 1930, when RVFV was first diagnosed in the Rift Valley of Kenya, numerous outbreaks of RVFV have been reported in many regions across Africa [4,5]. RVFV became a global concern in 1977–1978, when an epidemic was recorded in Egypt, and an estimated 200,000 human infections resulted in 600 deaths [6,7]. Thereafter, periodic outbreaks of RVFV infections were reported in Kenya, Tanzania and Zambia [6,8]. Sporadic outbreaks have also been reported throughout the African continent: Somalia (2006–2007); Kenya (2006–2007); Tanzania (2007); Sudan (2007–2008); Madagascar (2008–2009); South Africa (2008–2010); Mauritania (2009–2011); Botswana (2010); and Namibia (2010) [9]. The 2000 spread to Saudi Arabia and Yemen made RVFV a global concern [10,11,12,13,14,15,16]. RVFV outbreaks resulted in severe economic burden, especially in Africa where agriculture of livestock is a primary source of income for many livestock farmers. The loss of production and productivity during outbreaks has direct economic impact and can result in overall food shortages. The RVFV outbreak in East Africa between 2006 and 2007 resulted in the economic loss estimated to exceed $60 million [17].

RVF viral proteins and nucleic acids have been used as biomolecules for the development of diagnostic assays, such as the enzyme-linked immunosorbent assay (ELISA) and reverse transcriptase Polymerase Chain Reaction (RT-PCR) [18,19,20]. For RVF viral proteins diagnosis, serological techniques have been developed. Serological tests, namely, virus neutralization assay (VNA), ELISA and haemagglutination inhibition (HI), are designed to detect specific antibodies produced in infected animals or viral proteins that can function as antigens. The ELISA technique offers a reliable, safe and efficient tool for the detection of antibodies against RVFV, and together with RT-PCR and virus isolation by cell culture are to this end deemed acceptable for RVFV testing by the World Health Organisation (WHO) [10].

Although recognised and accepted by the WHO as definitive diagnostic tools for RVFV, these diagnostic assays require highly specialised infrastructure and trained personnel which are beyond the resources and capabilities of many laboratories in developing countries such as those in the sub-Saharan Africa [21]. This is especially problematic when outbreaks occur in remote regions and veterinary medical workers are unable to provide rapid diagnostic testing. A successful development of a point-of-care (POC) RVFV diagnostic device, that can be applied by veterinarians, abattoir and other high-risk workers who work with blood and tissue samples from animals, will contribute to improving the surveillance for RVFV epidemic, which will, in turn, have a positive impact in the control of RVFV.

Previous studies on immunochromatographic methods have demonstrated the feasibility of developing a lateral flow assay (LFA) for RVFV diagnosis [22,23]. In a study by Cêtre-Sossah et al., a first-line LFA for the detection of the RVFV N protein was developed and validated [23]. The LFA made use of immobilised monoclonal antibodies (MAbs) against the RVF N protein (produced in mice) on the test line to capture N protein present in the test samples. Another set of anti-RVFV N MAbs were conjugated to gold nanoparticles (AuNPs) to bind N proteins captured on the strip, with the AuNPs producing a visual signal. The control line, on the other hand, consisted of an anti-mouse antibody to capture excess antibody-AuNP conjugates and also produce the quality control line for the validation of the test [23].

In a different study by Sayed and colleagues, a one-step LFA that was able to detect RVFV IgM and IgG antibodies in sheep sera using an anti-IgM antibody and staphylococcus protein A (ProA) as capture reagents, respectively, was developed [22]. This study exploited the high affinity of ProA for IgG molecules and used ProA to capture these molecules, as opposed to an anti-IgG antibody, while an AuNP-conjugated RVFV antigen was used to detect captured RVFV antibodies.

This paper presents an approach similar to that of Sayed et al. for the immunochromatographic detection of RVFV antibodies. In the current study, however, ProA was used as both a capturing and detection reagent. This approach was to fully maximise on ProA’s ability to bind IgG molecules from different animal species and to completely bypass the use of species–specific antibodies, to potentially develop a device that could detect antibodies produced in different animal species infected with the RVFV. The non-species specificity of the device proposed herein could, therefore, be an added advantage for managing RVFV during outbreaks, and could also be more practical in disease surveillance programs where large numbers of samples from various animal species are screened, thus further assisting efforts to control the spread of the virus.

## 2. Methods

### 2.1. RVFV Recombinant Nucleocapsid Protein Biomarker Development

The full-length sequence of the gene encoding the RVFV nucleocapsid (Genbank accession number NC_014395.1) cloned into pET-32a expression vector by GenScript (Piscataway, NJ, USA) was transformed into competent *E. cloni*^®^ EXPRESS BL21 Competent Cells (Lucigen, Middleton, WI, USA) for expression of the RVFV recombinant nucleocapsid protein (rNp) by following the manufacturer’s instructions. Glycerol stocks of the expressed bacterial cells were prepared and stored at −80 °C.

The protocol of Jasen van Vuren et al., was adopted, with modifications, for the expression of the RVFV rNp [24]. Briefly, a bacterial culture was prepared by inoculating the LB Broth (Miller (Sigma-Aldrich, Darmstadt, Germany)) (Sigma-Aldrich (Merck)) containing ampicillin (Amp) (100 μg/mL) (Sigma-Aldrich (Merck)) with rNp glycerol stock. The resultant mixture was incubated overnight at 37 °C shaken at 200 rpm. The culture was upscaled by inoculating it (1:20) into fresh LB Amp and incubated at 37 °C and shaking until the optical density at 600 nm (OD_600_) reached 0.6–0.7. The expression of the rNp was induced by adding of isopropyl *β*-D-1-thiogalactopyranoside (IPTG) (1 mM) (Sigma-Aldrich (Merck), Darmstadt, Germany) and incubating at 37 °C with shaking at 200 rpm. Aliquots of the induced culture were sampled on an hourly basis for a total period of 5 h. The cells from the hourly collected aliquots were harvested by centrifugation for 10 min at 10,000× *g*, after which the supernatants were discarded and the pellets stored at −80 °C.

The harvested cell pellets were lysed using the BugBuster Protein Extraction Reagent (Novagen (Merck)) by following manufacturer’s instructions. Briefly, the cell pellets were resuspended in the BugBuster reagent and thereafter incubated for 20 min at room temperature on a shaking platform. The insoluble cell debris was removed by centrifugation at 4 °C at 10,000× *g* and the supernatants were analysed for the presence of the rNp protein using SDS PAGE analysis. For the SDS-PAGE analysis, the supernatants were treated with 1:20 β-mercaptoethanol laemmli, boiled for 5 min at 95 °C and then loaded on the 12% Mini-PROTEAN TGX Stain-Free Precast Gel (Biorad, Hercules, CA, USA). Electrophoresis was conducted on the loaded gel at 200 V in 1× Tris-Glycine-SDS (TGS) (25 mM Tris Base, 192 mM glycine, 0.1% sodium dodecyl sulfate) running buffer for 40 min. This was followed by the western blot analysis, where proteins from SDS-PAGE were transferred to the polyvinylidene difluoride (PVDF) membrane using the iBlot^®^ dry blotting system (Thermo Fischer, Waltham, MA, USA) along with the iBlot Transfer Stacks (Thermo Fischer, Waltham, MA, USA). The membrane was blocked overnight at 4 °C in a blocking solution consisting of Tris-buffered saline (TBS) (Sigma-Aldrich (Merck), Darmstadt, Germany) and 3% (*w*/*v*) Bovine serum albumin (BSA) (Sigma-Aldrich (Merck)). After washing the membrane in the wash solution (TBS-T, 0.05% (*v*/*v*) Tween 20 (pH 7.5)), the anti-RVFV nucleocapsid antibody produced in rabbit (rAb; Sigma-Aldrich (Merck), Darmstadt, Germany), was diluted to 1:10,000 in the blocking solution prior to being added to the PVDF membrane. The membrane was then incubated in the solution for 1 h at room temperature with gentle agitation. The membrane was washed as described above, followed by 1 h incubation with the anti-Rabbit IgG–Peroxidase antibody (Sigma-Aldrich (Merck)) and the StrepTactin HRP (Biorad, Hercules, CA, USA). diluted to 1:60,000 and 1:5000, respectively, in the blocking solution. Thereafter, the blot was washed, and the Clarity^TM^ western ECL substrate kit (Biorad) was used for the signal development. The blot was imaged using the Chemidoc imaging system (Biorad).

The expression of the rNp was upscaled to 2 L and was purified using a 5 mL HisTrap™ HP column (Global Life Sciences Solutions (Cytiva), Marlborough, MA, USA) with the NGC Chromatography Systems (Biorad). The column was equilibrated with the binding buffer before loading it with the rNp lysate. It was then washed sequentially with Wash Buffer 1 (50 mM Na_2_HPO_4_, 300 mM NaCl, 25 mM imidazole, pH 8.0) and Wash Buffer 2 (50 mM Na_2_HPO_4_, 300 mM NaCl, 60 mM imidazole, pH 8.0), and the protein was eluted with the elution buffer (50 mM Tris-HCl pH 8.0, 300 mM NaCl, 450 mM Imidazole). The eluted fractions were collected and analysed for the presence of the rNp protein via SDS-PAGE and western blot analysis. The elution fractions containing the rNp were pooled and concentrated into the storage buffer (50 mM Tris-HCl, 500 mM NaCl, 10% *v*/*v* glycerol, pH 8.0) using the Amicon^®^ Stirred Cell (Amicon Millipore (Merck)). The protein concentration was determined using a NanoDrop 2000 Spectrophotometer (Thermo Fischer, Waltham, MA, USA). The purified RVFV rNp was to be used as the antigen in the development of the of the RVFV lateral flow assay.

### 2.2. RVFV Lateral Flow Assay Development

#### 2.2.1. Biomarkers, Membranes and Pads

The Protein A (ProA), backing cards, nitrocellulose Whatman AE 98 fast membrane and Millipore GF041 conjugate pads were purchased from Diagnostic Consulting Network (DCN, Fremont, CA, USA). The nitrocellulose Whatman AE 98 fast membrane belongs to an AE family of membranes which are specifically manufactured for vertical-flow assays.

#### 2.2.2. Buffers

Various buffer solutions were prepared as follows: conjugate pad treatment buffer was composed of 10 mM borate, 3% BSA, 1% polyvinylpyrrolidone-40 (PVP-40), 0.25% Triton X-100 at pH 8. The membrane blocking buffer consisted of 10 mM sodium phosphate, 4% sucrose, 0.1% BSA, 0.075% PVP-40 at pH 7.4. The dilution buffer was made up of 1% BSA in PBS. The conjugate diluent Buffer contained 50 mM Borate, 1% BSA at pH 9. The conjugate blocking buffer 1 consisted of 50 mM borate, 10% BSA, pH 9, while the conjugate blocking buffer 2 was made up of 0.1% polyethylene glycol (PEG). The running buffer 1 consisted of 1% BSA and the running buffer 2 of 0.1% Triton X-100 (1%) BSA.

#### 2.2.3. Gold Nanoparticle Synthesis

The method for the synthesis of the colloidal AuNPs followed a well-established Turkevich method [25]. Briefly, 40 M C_6_H_5_Na_3_O_7_.2H_2_O was used to reduce a solution of 30 M HAuCl_4_·3H_2_O at 100 °C and under stirring conditions. The resulting reaction mixture changed from grey to dark purple and thereafter to wine-red. The wine-red solution was removed from the heat and allowed to continue stirring at room temperature for a further 3 h. The resultant AuNPs were characterised using the Thermo Scientific Multiskan GO UV-Vis Microplate Spectrophotometer (Thermo Fischer, Waltham, MA, USA) to collect their absorption spectra, and Jeol 2100 transmission electron microscope (TEM) (Tokyo, Japan) to study their morphology.

#### 2.2.4. Preparation of the ProA-AuNPs Detection Conjugate

The procedure for the preparation of the ProA-AuNP detection conjugate was adopted with modification from Mdluli et al. [26]. In a typical procedure, a volume of 100 µL of 1 mg/mL ProA solution was added to 14 nm AuNPs (10 mL, pH 8, Optical density (OD) 1) at ambient temperature and incubated for 15 min with gentle shaking. To remove any unbound ProA, the reaction mixture was centrifuged at 15,000 rpm for 15 min and 4 °C. The AuNP pellet was resuspended in a blocking buffer solution of 10% BSA in 50 mM Borate and thereafter incubated for 30 min at room temperature under gentle shaking conditions. The contents of the reaction mixture were centrifuged for 15 min at 15,000 rpm and 4 °C. The supernatants from the conjugated pellet were aspirated and the pellet was resuspended with a conjugate diluent buffer (50 mM Borate, 10% BSA, pH 9) to an OD of 10.

#### 2.2.5. Preparation of Conjugate Pads

The conjugate pads were first cut 35 mm long to fit the “conjugate pad” part on the Backing Card. The pads were then blocked using the conjugate block buffer (10 mM Borate, 3% BSA, 1% PVP-40, 0.25% Triton X-100 (pH 8)) and thereafter dried for 45 min at 37 °C.

#### 2.2.6. Assembly of the Strip 

**Figure 1:**
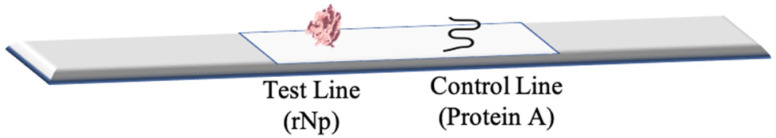
The configurations of the RVFV strip assembly.

The nitrocellulose membrane was first assembled onto the plastic backing support. Purified capture rNp (5 mg/mL) and ProA (0.5 mg/mL) were immobilised at a rate of 1 µL/cm onto the membrane using the Biodot XYZ Series dispensing system, forming the test and control lines, respectively. The membrane was subsequently dried at 37 °C for 15 min. The dried blocked conjugation pad was then assembled onto the backing card allowing for 1–2 mm overlap onto the membrane on the side of the test line. The absorbent pad was attached to the support at the other end of the membrane with a 1–2 mm overlap. The absorbent pad was then cut into 5-mm thick strips coated with 0.05 µL of 5 mg/mL rNp on the test line and 0.05 µL of 0.5 mg/mL ProA on the control line. The strips were stored at room temperature in a sealed microarray storage aluminium pouch. The configuration of the assembled half-strip is shown in Figure 1. This half-strip configuration is necessary for the early stages of LFA development.

#### 2.2.7. The Dipstick (Half-Strip) Liquid Conjugate Testing

**Figure 2:**
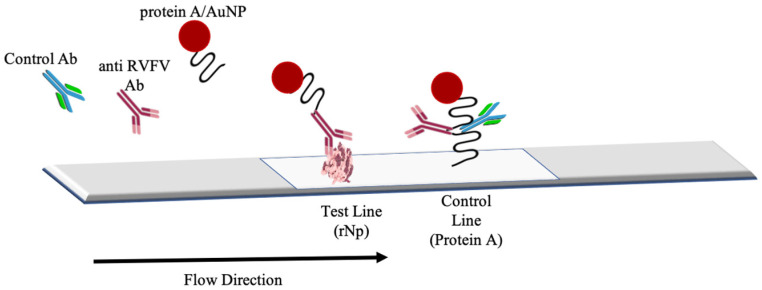
An illustration of RVFV lateral flow strip for a positive test.

The ability of the ProA-AuNP detection conjugate to bind to the test and the control lines on the RVFV half-strip was tested using the dipstick method. The test samples were prepared by adding the anti-RVFV rAb to different final concentrations (0.1, 0.125, 0.15, 0.2 and 0.4 mg/mL), 5 µL of ProA-AuNP conjugate, in addition, 5 µL of anti-Rabbit IgG secondary Ab (control antibodies) and PBS buffer to a final volume of 20 µL. The mixture allowed for the formation of the anti-RVFV rAb-ProA-AuNP conjugate and of the anti-Rabbit IgG-ProA-AuNP conjugate complexes. The RVFV half-strip was dipped into the mixtures and the liquid migrated through the membrane. The results were interpreted after allowing the test to run until the flow reached the absorbent pad. The anti-RVFV rAb on the anti-RVFV rAb-ProA-AuNP conjugate complex binding to the capture rNp antigen on the membrane and forming a red-coloured band at the test line characterises a positive test result. The control Ab binds to ProA-AuNP conjugate to form an anti-Rabbit Ab/AuNP/antibody complex, which interacts with the immobilised ProA on the control line and is characterised by the red band visualised on the test. In the absence of the anti-RVFV rAb, only the control line will appear on the strip. The reaction on the control line indicates the proper flow of the analytes using the PBS running buffer. Figure 2 illustrates the RVFV lateral flow strip positive test after being subjected to the liquid-conjugate test. The RVFV half strips were also tested using the RVFV IgG-positive bovine serum containing bovine RVFV antibodies (anti-RVFV bAb) (IDvet, Grabels, France) and the negative bovine serum (control Ab) (IDvet, Grabels, France) which are supplied as an internal reference material for quality control of the ID Screen^®^ Rift Valley Competition Multi-species ELISA kit.

## 3. Results

### 3.1. RVFV rNp Biomarker Development

**Figure 3:**
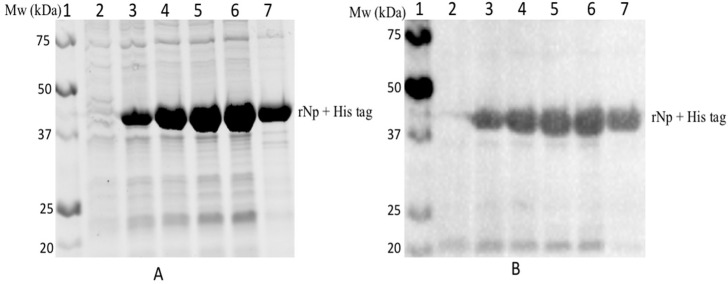
SDS-PAGE (**A**) and western blot (**B**) of the bacterially expressed RVFV rNp. SDS-PAGE (**A**) and western blot (**B**) of the bacterially expressed RVFV rNp. Lane 1: Precision Plus Protein Unstained Standard marker, lane 2: uninduced lysate and lane 3 to lane 7: IPTG induced lysate collected hourly from 1 to 5 h.

Figure 3 shows that the transformation of the E. cloni^®^ EXPRESS *E. coli* cells with the pET32a plasmid containing the RVFV nucleocapsid genetic sequence resulted in an over-expression of the 45 kDa rNp fusion protein. The SDS-PAGE gel (Figure 3A) shows that the expressed rNp falls within the expected size of 45 kDa. Also evident from Figure 3A is that the optimum expression of the rNp protein occurred 4 h after induction with IPTG. Lane 2 shows the un-induced sample and as such no expression occurred. A decrease in the rNp expression levels was observed after 5 h of induction (Lane 7). The western blot analysis using anti-RVFV rAb confirmed the successful expression of the RVFV rNp protein (see Figure 3B).

**Figure 4:**
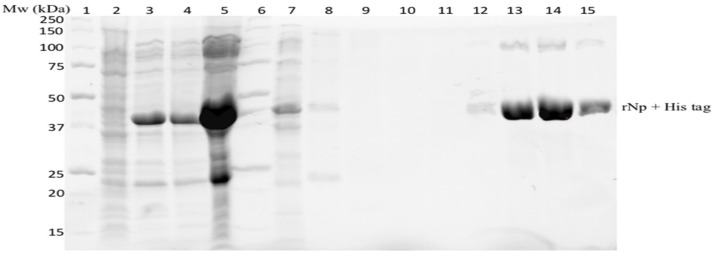
SDS-PAGE of the expressed and purified RVFV rNp. Lanes 1 and 6: Precision Plus Protein Unstained Standard marker, lane 2: uninduced bacterial lysate, lane 3: cell lysate after 4 h induction, lane 4: rNp pellet, lane 5: rNp supernatant, lane 7: flow-through HisTrap HP columns, lanes 8–10: washes and lanes 12–15 eluted rNp protein.

*E. coli* cells that showed successful expression of the RVFV rNp protein were lysed and purified using nickel affinity chromatography. In Lane 3 of Figure 4, an overexpression of the rNp in the lysate after 4 h induction and IPTG to a final concentration of 1 mM at 37 °C is observed. The rNp was observed mainly in the soluble fraction (Lane 5). As shown in Figure 4, most of the unwanted protein species that were co-expressed with rNp did not bind the nickel affinity column and were mostly present in the flow-through observed in lane 7, while rNp remained bound to the column. The protein was eluted using a linear imidazole gradient from 60 mM to 500 mM. It is quite evident from the eluted fractions displayed in Lanes 12–15 that the purification of rNp was achieved. The purified rNp was used as the capture antigen for the LFA.

### 3.2. RVFV Lateral Flow Assay Development

**Figure 5:**
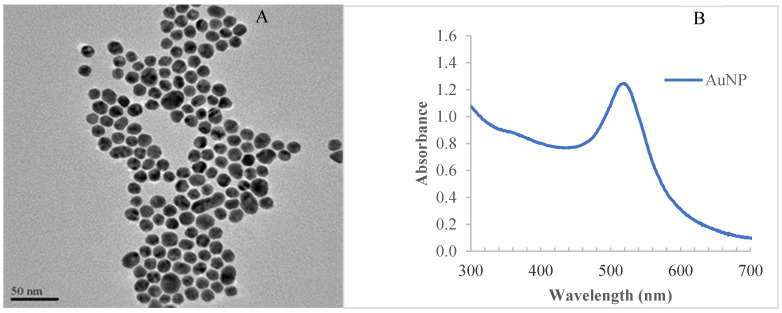
(**A**) A TEM micrograph of the AuNPs. (**B**) The spectra of the AuNPs by UV–visible spectrophotometry.

RVFV LFA development entails the synthesis and characterisation of AuNP and strip development. The size of the synthesised AuNPs measured from the (TEM) micrographs were 14 ± 2 (see Figure 5A). The AuNPs were characterised using UV–Vis spectrophotometry to monitor the absorption spectra is shown in Figure 5B. The localised surface plasmon resonance (LSPR) of 14 nm AuNPs resulted in a strong absorbance band in the visible region of 519 nm. The architecture and testing mechanism of the strip is discussed subsequently.

**Figure 6:**
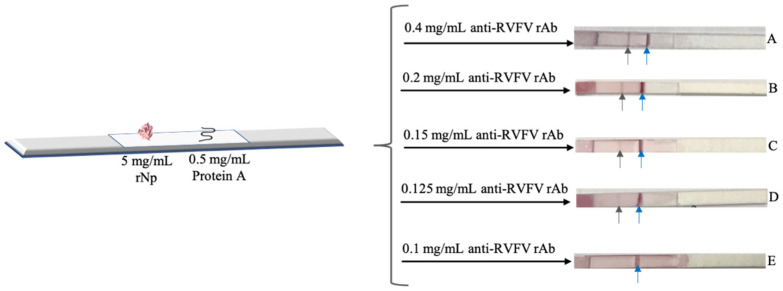
Assembled RVFV lateral flow assay strips testing the limit of detection with 5 mg/mL rNp on test line and 0.5 mg/mL ProA on the control line tested with various concentrations of anti-RVFV rAb. (**A**–**E**) shows the LFA results after testing with anti-RVFV rAb concentrations of 0.4, 0.2, 0.15, 0.125 and 0.1 mg/mL respectively. The black and blue arrows denote the test control lines, respectively.

This study investigated a half-strip format. The half-strip consists of a small portion of the conjugation pad, nitrocellulose membrane and the absorbent pad. The RVFV lateral flow assay half-strip is deemed necessary for the early stages of LFA development, such as reported herein. The results presented in Figure 6A–D showed the appearance of both the test line (T) band and the control line (C) band in the half-strip. This indicates the proper flow of the analytes. Furthermore, a decrease in the intensity of the band was observed when the concentration of anti-RVFV rAb was decreased. As shown in Figure 6E, the concentration of 0.1 mg/mL was not detected by the 5 mg/mL rNp strip. The lowest limit of detection of RVFV Np antibodies by the 5 mg/mL rNp test line and 0.5 mg/mL control line RVFV lateral flow strip was found to be 0.125 mg/mL.

**Figure 7:**
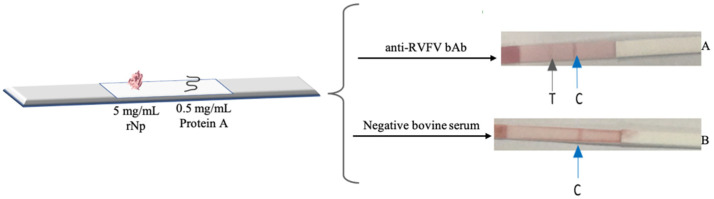
The liquid-test results on the investigation of the RVFV positive (**A**) and negative (**B**) sera. The black and blue arrows denote the test and control line, respectively.

The RVFV IgG-positive bovine serum was used to check the ability of the RVFV half-strip to detect the anti-RVFV bAb in a serum sample. A negative bovine serum (IDvet, Grabels, France), which does not contain any antibodies against RVFV, was used as a negative control to check for specificity of the half-strip. The RVFV half-strip was able to detect the RVFV bovine IgG antibodies in the serum, as evidenced by the appearance of the test and control lines indicated by the red and the blue rows, respectively, in Figure 7A.

## 4. Discussion

### RVFV Lateral Flow Assay Development

The observed size of 14 ± 2 nm AuNP is indicative of a narrow particle size distribution. This is ideal for a good label material. While most LFAs use 40 nm, it was a desirable choice to explore smaller sized NPs for fabrication of the RVFV LFA. The idea was to exploit the bright red colour and stability presented by smaller sized AuNPs, e.g., 10–20 nm. The larger AuNPs presents the desirable larger surface areas for bioconjugation but lacks stability and colour intensity [27].

The RVFV half-strip that was developed in this study was successful in detecting purified RVFV antibodies. Furthermore, the ability of the half-strip to detect RVFV antibodies from bovine serum was also tested. The RVFV half-strip format could detect the RVFV bovine IgG antibodies in the serum; however, no degree of selectivity could be established. The RVFV IgG-positive bovine serum was only supplied as an internal reference material for quality control of the ID Screen^®^ Rift Valley Competition Multi-species ELISA kit; as such, details on how the serum was prepared was not provided. However, the degree of intensity of the test line in Figure 7A, was comparable to that in Figure 6D. It can, therefore, be estimated that the concentration of the anti-RVFV bAb in the serum is approximately 0.125 mg/mL. Furthermore, the control line observed for the negative RVFV serum in Figure 7 appeared less intense compared to the rabbit IgG Ab control line in Figure 6. This could be due to the lower affinity of ProA for bovine IgG than rabbit IgG [28].

Reports about the ProA’s ability to bind to the Fab domains of some IgM and IgA molecules, but with lesser affinity than that reported for IgG, have also been published [29,30]. The above-mentioned properties have made ProA an attractive component in biotechnology applications, particularly with applications that involve purification and detection of antibodies [31]. The approach of using ProA as a detection and capturing reagent, which was adopted in this study, has been investigated and reported several times in the development of serological diagnostic tools such as LFA and ELISA for various pathogens [32,33,34].

Appropriate validation studies using field samples from various host species to validate the performance of the LFA device would, however, be recommended prior to its application for diagnostic and surveillance programs. The preliminary study addresses the ability of the expressed rNp to detect the targeted anti-RVFV antibodies when immobilised on the LFA platform using ProA on the detection conjugate and on the control line. Specific and deliberate efforts were directed towards demonstrating a proof-of-concept for the assay. A future study will further investigate the sensitivity and selectivity performance of the assays towards the RVFV serum. 

## 5. Conclusions

A functional diagnostic device relies on the binding events between the target analyte and its biorecognition molecule and the conversion of this reaction into a detectable signal. In this study, a proof-of-concept of the development of the lateral flow assay for RVFV diagnosis capable of detecting anti-RVFV antibodies in pure antibody samples and RVFV bovine sera has been demonstrated. Specifically, the functioning of the different components of the LFA, such as the antibody-antigen binding on the LFA membrane and the ProA-AuNP detection conjugation, have been demonstrated. The format used in the LFA of this study consisted of a capture antigen (RVFV rNp) and ProA immobilised on a nitrocellulose membrane forming the test and control lines, respectively. The strategy of using ProA as a capturing and detection reagent proved to be successful, as ProA was able to bind antibodies in the analyte, which, in turn, bound to the ProA immobilised on the control line. This approach has the potential to eliminate the use of species-specific antibodies and can, therefore, be modified to detect antibodies from various animal species. The developed RVFV half-strip is able to detect RVFV antibodies with a limit of detection of 0.125 mg/mL.

At the time of undertaking of this work, our institution lacked the requisite approval from government authorities to obtain samples of RVFV serum for testing. As a result, a significant limitation of this study is the inability to fully evaluate the performance of the prototype assay in clinical samples. Future work should include optimisation of the detection conjugate, investigating how increasing the concentration of the coated rNp and ProA affect the sensitivity of the assay, testing on positive and negative multispecies sera to determine the selectivity and specificity of the rNp towards the targeted RVFV antibodies, investigating additional components that are required for the development of an RVFV full-strip into a working prototype and establishing rapidity of the LFA that is compatible with the standard. The selectivity and sensitivity of the working RVFV LFA prototype should also be compared to the existing RVFV tests such as the ID Screen^®^ Rift Valley Competition Multi-species ELISA test.

## Data Availability

The data displayed in this work is available on request from the corresponding author.
